# Supplementary material for: MicroRNA Analysis of Human Stroke Brain Tissue Resected during Decompressive Craniectomy/Stroke-Ectomy Surgery
Source: Genes (Basel). 2021 Nov 23;12(12):1860. doi: 10.3390/genes12121860 (PMC8702168; doi:10.3390/genes12121860)
Supplement: Supplementary file 1 [file genes-12-01860-s001.zip › genes-1437989-supplementary.pdf]

Supplemental Materials

Supplemental Tables S1 and S2
Online Supplemental Figures
S1-S3 References: 52-61

Supplemental Table S1, stroke and non-stroke control patient samples used for analyses. ICA - internal carotid artery; MCA - middle cerebral artery; LKN - last known normal time; L - left; R - right.

Stroke patients	Artery involved	Samples collected from	Time after stroke onset/ LKN	Age	Sex
S1	L ICA	L temporal lobe	48 hours	57	F
S2	R ICA	R temporal lobe	72 hours	23	M
S3	R MCA	R temporal lobe	17 hours	59	M
S4	L MCA	L temporal lobe	28 hours	50	M
S5	R MCA	R temporal lobe	60 hours	38	M

Non-stroke controls	Samples collected from	Diagnosis of tissue	Diagnosis of case	Age	Sex
C1	R temporal lobe	Normal	Oligoastrocytoma	44	M
C2	L temporal lobe	Normal	Glioblastoma	64	M
C3	R temporal lobe	Normal	Glioblastoma	52	F

Supplemental Table S2, stroke-related miRNAs dysregulated in human stroke brain tissue

Name	Fold-change	P-value	FDR P-value	Reported in:
hsa-miR-1246	334.46	0	0	[40-42]
hsa-miR-4516	32.68	0	0	[43]
hsa-miR-182-5p	15.44	3.98E-06	0.0002	[18]
hsa-miR-183-3p	11.02	0.0207	0.2654	[18]
hsa-miR-320d	10.80	5.73E-11	8.67E-09	[44,48]
hsa-miR-1255b-5p	9.53	0.0001	0.0059	[49]
hsa-miR-320c	9.29	2.30E-11	3.79E-09	[44]
hsa-miR-183-5p	8.87	0.0001	0.0059	[18]
hsa-miR-96-5p	8.45	0.0004	0.0144	[18]
hsa-miR-196b-5p	5.64	0.0011	0.0347	[18]
hsa-miR-15b-5p	3.54	0.0025	0.0611	[45]
hsa-miR-1299	3.46	0.0163	0.2278	[18,41]
hsa-miR-18a-5p	3.33	0.0026	0.0628	[41]
hsa-miR-204-3p	3.23	0.0008	0.0251	[46]
hsa-miR-17-5p	3.20	0.0005	0.0180	[45]
hsa-miR-106b-3p	3.12	0.0032	0.0720	[41]
hsa-miR-224-5p	3.11	0.0028	0.0666	[41]
hsa-miR-101-2-5p	3.07	0.0394	0.3912	[50,51]
hsa-miR-15b-3p	3.07	0.0186	0.2540	[45]
hsa-miR-18a-3p	2.91	0.0303	0.3438	[41]
hsa-miR-193b-5p	2.87	0.0004	0.0142	[51]
hsa-miR-483-5p	2.83	0.0153	0.2164	[14]
hsa-miR-629-3p	2.83	0.0173	0.2393	[18]
hsa-miR-16-5p	2.78	0.0002	0.0090	[47]
hsa-miR-18b-5p	2.73	0.0350	0.3741	[41]
hsa-miR-187-5p	2.70	0.0401	0.3954	[18]
hsa-miR-484	2.60	0.0025	0.0610	[47]
hsa-miR-106b-5p	2.59	0.0221	0.2770	[41]
hsa-miR-20a-5p	2.56	0.0116	0.1902	[50]
hsa-miR-1908-5p	2.55	0.0394	0.3912	[52]
hsa-miR-320b	2.54	0.0017	0.0465	[44]
hsa-miR-574-5p	2.53	0.0082	0.1443	[18]
hsa-miR-106a-5p	2.47	0.0146	0.2164	[41]
hsa-miR-423-5p	2.46	0.0015	0.0416	[41]
hsa-miR-320a-3p	2.41	0.0008	0.0263	[44]
hsa-miR-21-5p	2.39	0.0071	0.1329	[53]
hsa-miR-502-3p	2.37	0.0016	0.0455	[18]
hsa-miR-505-5p	2.34	0.0014	0.0416	[18]
hsa-miR-576-3p	2.34	0.0288	0.3309	[18]
hsa-miR-129-1-3p	2.24	0.0085	0.1484	[18]
hsa-miR-93-5p	2.21	0.0346	0.3710	[18]
hsa-miR-652-3p	2.19	0.0015	0.0416	[18]

hsa-miR-194-5p	2.12	0.0228	0.2775	[54]
hsa-miR-877-5p	2.11	0.0048	0.0987	[48]
hsa-miR-92a-3p	2.04	0.0226	0.2775	[45]
hsa-miR-574-3p	2.04	0.0064	0.1240	[18,55]
hsa-miR-501-3p	2.03	0.03370	0.3641	[18]
hsa-let-7d-3p	2.01	0.01899	0.2554	[18]
hsa-miR-625-5p	1.99	0.01113	0.1854	[18]
hsa-miR-193a-5p	1.97	0.02501	0.2987	[51]
hsa-miR-27a-3p	1.95	0.02750	0.3199	[50]
hsa-miR-146b-3p	1.89	0.02587	0.3069	[56]
hsa-miR-941	1.88	0.03576	0.3753	[48]
hsa-miR-193b-3p	1.84	0.033228	0.3633	[51]
hsa-miR-184	1.81	0.026221	0.3090	[14]
hsa-miR-362-5p	1.80	0.032322	0.3577	[57]
hsa-miR-155-5p	1.73	0.041987	0.4119	[57]
hsa-miR-625-3p	1.68	0.047847	0.4408	[18]
hsa-miR-376a-2-5p	-1.51	0.070770	0.5359	[58]
hsa-miR-29a-5p	-1.67	0.045963	0.4300	[14,42]
hsa-let-7f-2-3p	-1.69	0.048431	0.4422	[59]
hsa-let-7c-3p	-1.73	0.022499	0.2775	[18,42]
hsa-miR-9-5p	-1.76	0.002994	0.0697	[60]
hsa-miR-135a-5p	-1.79	0.037469	0.3821	[18]
hsa-miR-409-5p	-1.82	0.028192	0.3260	[58]
hsa-miR-218-5p	-1.88	0.009462	0.1620	[50]
hsa-miR-455-5p	-1.90	0.018269	0.2512	[61]
hsa-miR-7-5p	-2.03	0.002281	0.0587	[59,18]
hsa-miR-106a-3p	-2.07	0.019659	0.2604	[41]
hsa-miR-376c-5p	-2.10	0.004647	0.0980	[58]
hsa-miR-432-3p	-2.20	0.048482	0.4422	[58]
hsa-miR-542-3p	-2.39	0.003690	0.0817	[61]
hsa-miR-135a-3p	-9.00	0.000536	0.0180	[18]
hsa-miR-196a-3p	-17.60	0.003520	0.0789	[18]
hsa-miR-196a-5p	-31.65	3.33E-16	1.01E-13	[18]

Supplemental Figure S1

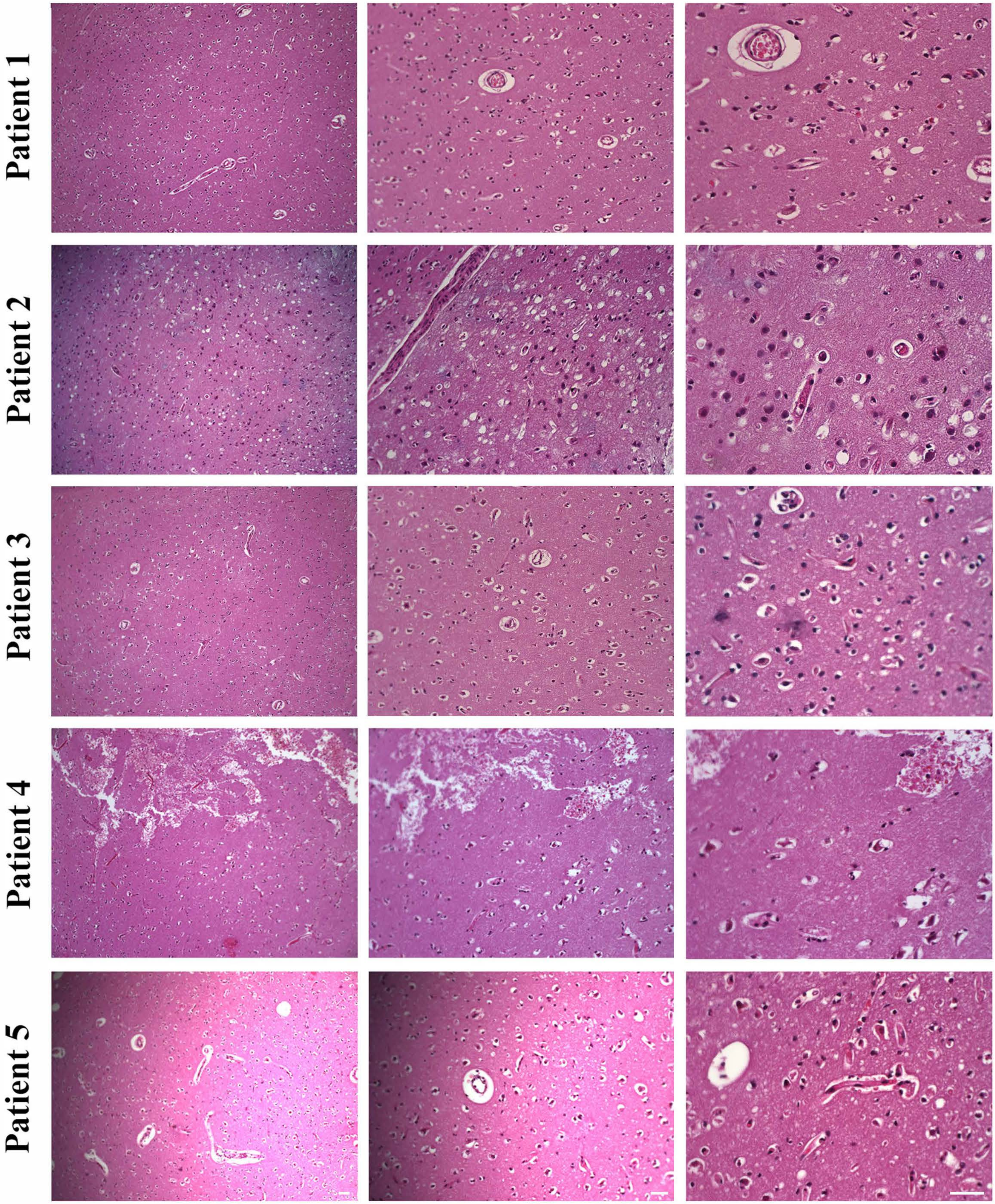

Supplemental Figure S2

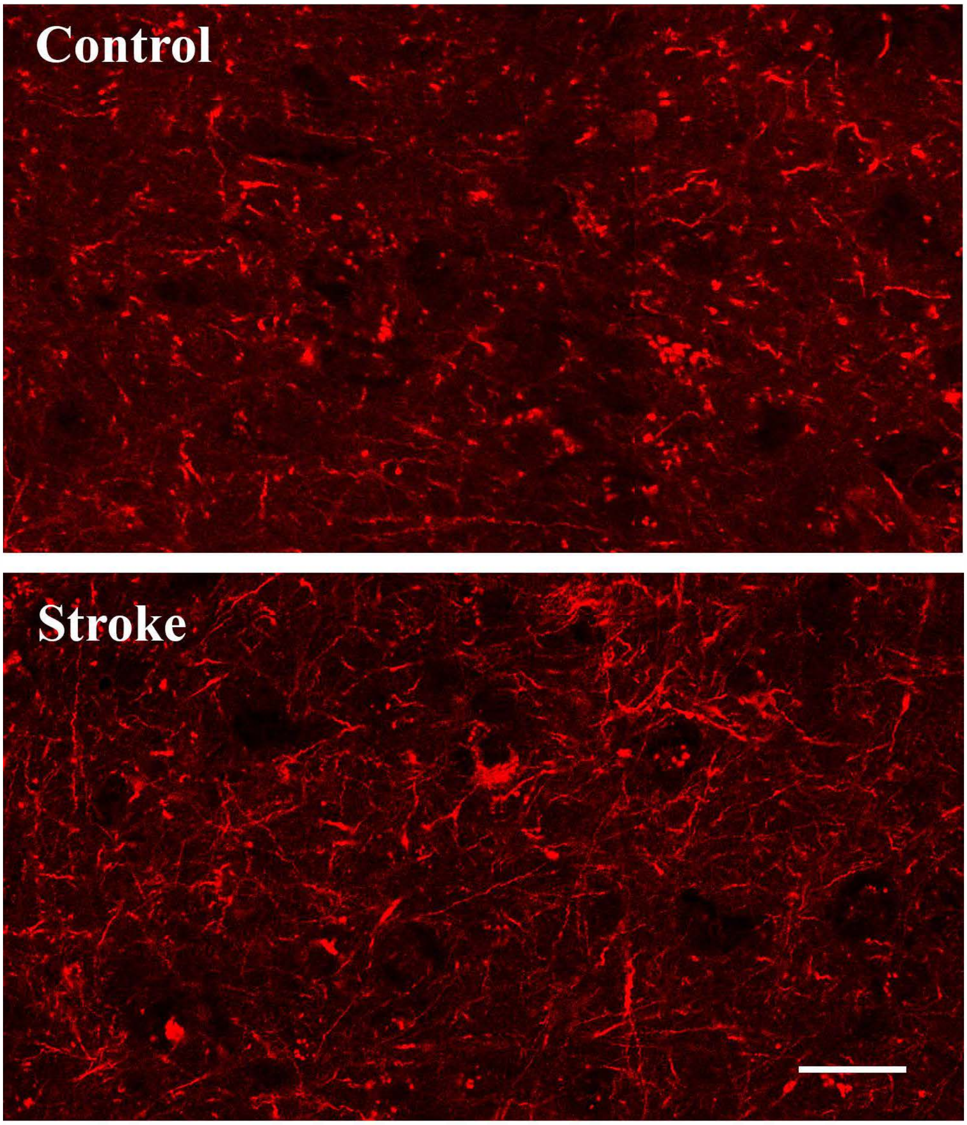

Supplemental Figure S3

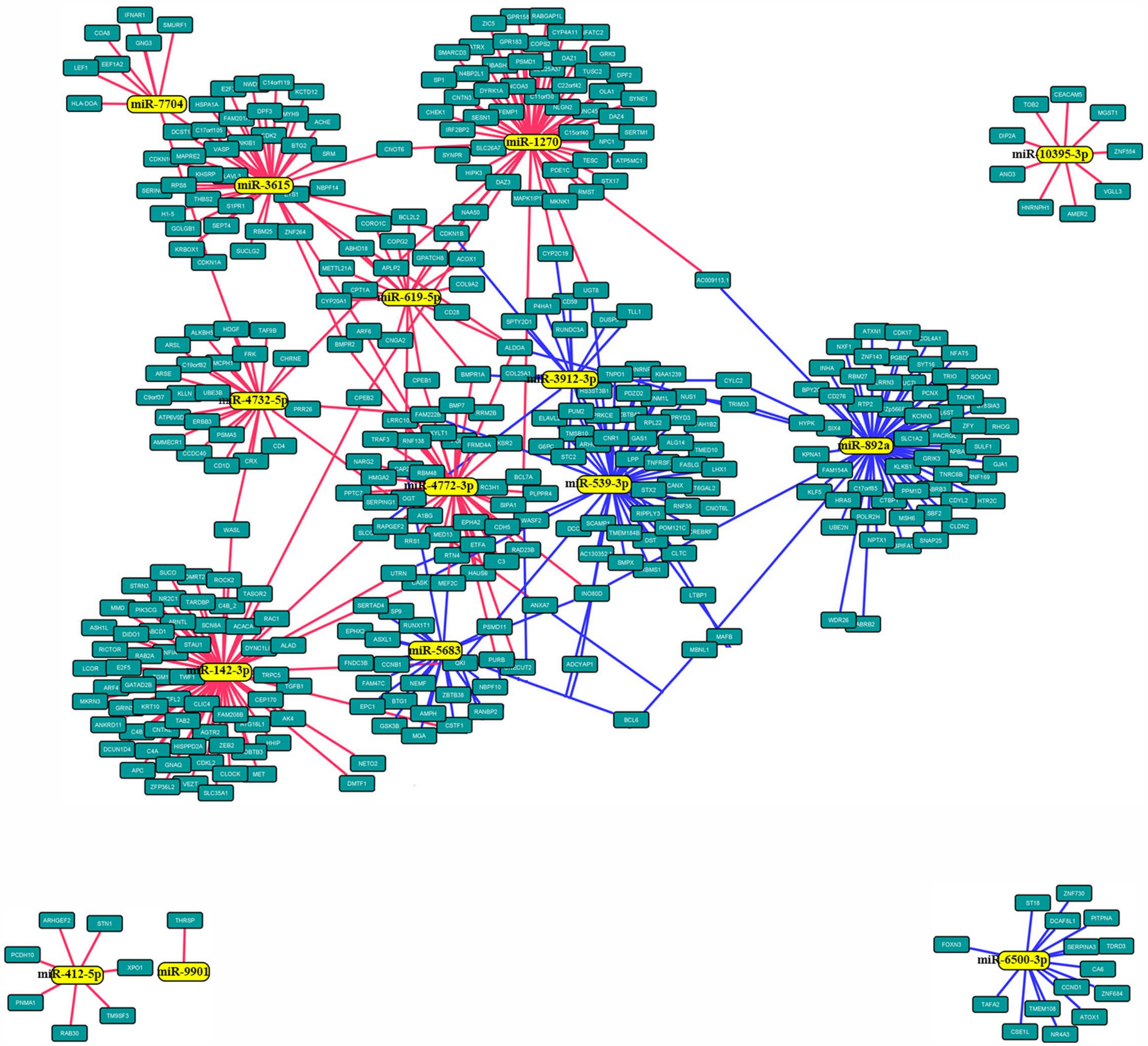

Online Supplemental Figure Legends

Supplemental Figure S1. Morphology of human stroke brain temporal lobe tissue

Representative images of the tissue from 5 different stroke patients, 10x, 20x, and 40x magnifications. HE staining. Bars: 50 µm.

Supplemental Figure S2. Visualization of neuronal network in human brain tissue

Immunofluorescence staining with pan-neuronal marker antibody, temporal lobe tissue. A: non-stroke control sample; B: stroke tissue sample. Bar: 50 µm.

Supplemental Figure S3. Target genes regulated by newly identified dysregulated miRNAs

Diagram demonstrating newly detected significantly differentially expressed miRNAs and selected experimentally verified and predicted target genes. Note that number of miRNAs share the common target genes. Lines show the connections between the upregulated (red lines) and downregulated (blue lines) miRNAs with their target genes.
